# Tuberculosis in Lemurs and a Fossa at National Zoo, Madagascar, 2022

**DOI:** 10.3201/eid2912.231159

**Published:** 2023-12

**Authors:** Marni LaFleur, Hoby A. Rasoanaivo, Tojo H. Andrianarivo, Fanantenana Randria Andrianomanana, Stuart McKernan, Mamy Serge Raherison, Rakotoson Andrianantenaina, Michele Miller, Jonah Ratsimbazafy, Simon Grandjean Lapierre, Paulo Ranaivomanana, Niaina Rakotosamimanana

**Affiliations:** University of San Diego, San Diego, California, USA (M. LaFleur);; Lemur Love Inc., San Diego (M. LaFleur, H.A. Rasoanaivo);; University of Antananarivo, Antananarivo, Madagascar (H. Rasoanaivo, T.H. Andrianarivo);; Pasteur Institute of Madagascar, Antananarivo (T.H. Andrianarivo, F.R. Andrianomanana, M.S. Raherison, P. Ranaivomanana, N. Rakotosamimanana);; Wildlife One Health, Mtunzini, South Africa (S. McKernan);; Programme National de Lutte Contre la Tuberculose, Antananarivo (M.S. Raherison);; University of Stellenbosch, Cape Town, South Africa (M. Miller);; Centre de Recherche du Centre Hospitalier de l’Université de Montréal, Montréal, Quebec, Canada (S. Grandjean Lapierre);; Université de Montréal, Montréal, Canada (S. Grandjean Lapierre)

**Keywords:** tuberculosis, lemurs, fossa, Madagascar, tuberculosis and other mycobacteria, bacteria, primate, anthropozoonotic, Mycobacterium tuberculosis

## Abstract

We diagnosed *Mycobacterium tuberculosis* in captive lemurs and a fossa in Antananarivo, Madagascar. We noted clinical signs in the animals and found characteristic lesions during necropsy. The source of infection remains unknown. Our results illustrate the potential for reverse zoonotic infections and intraspecies transmission of tuberculosis in captive wildlife.

In 2020, the World Health Organization estimated that in Madagascar there were 238 cases of *Mycobacterium tuberculosis* complex infection per 100,000 persons, but fewer than half of infected persons were given the appropriate diagnosis and notification from public health authorities. Madagascar healthcare and wildlife protection sectors have low efficacy because government programs and infrastructure often face insufficient funding, ineffective initiative implementation, and corruption (*1*).

Captured wild animals experience high levels of stress and are often malnourished, which leads to immunosuppression (*2*). Most of the zoos in Madagascar, including the country’s national zoo, the Botanical and Zoological Park of Tsimbazaza (PBZT), hold wild-captured wildlife and have substandard captive care compared with zoos in developed countries (*3*,*4*). Humans, who may harbor pathogens, and animals at captive facilities in Madagascar are in close contact, creating an ideal setting for zoonotic and reverse zoonotic disease transmission (*5*).

We previously reported *M. tuberculosis* (lineage 3 with streptomycin resistance) infection in a wild-captured, pet ring-tailed lemur (*Lemur catta*) in Madagascar (*6*). This disease has not been reported in wild lemurs, although few populations or species have been screened (*7*). In this report, we document *M. tuberculosis* infection in *L. catta* and several other threatened species of lemurs, as well as in a fossa (*Cryptoprocta ferox*), in Madagascar (Table; Appendix Table). 

**Table:** Details on *Mycobacterium tuberculosis* complex–positive animals at PBZT, Madagascar, 2022*

Animal	Species (animal number)	Sex	Birth location	Date of birth	Date of death	Sample method	*M. tuberculosis* detected by GeneXpert	*M. tuberculosis* sublineage
Fossa	*Cryptoprocta ferox*	M	Wild	Unknown	2022 Aug	Necropsy	Low	4.3.3
Lemur	*Eulemur flavifrons*	F	Unknown	Unknown	Alive in 2022 Oct	Swab	Trace	4.3.3
	*E. fulvus*	M	Unknown	Unknown	Alive in 2022 Oct	Swab	Trace	4.3.3
	*E. fulvus*	F	Unknown	Unknown	Alive in 2022 Oct	BAL	Trace	4.3.3
	*E. rufus*	F	Unknown	Unknown	Alive in 2022 Oct	Swab	Trace	4.3.3
	*Lemur catta*	F	Unknown	Unknown	Alive in 2022 Oct	BAL	Trace	4.3.3
	*Propithecus coquereli*	M	Wild	Unknown	Early 2022	Necropsy	Low	4.3.3
	*Varecia variegata* (1)	F	PBZT	2019 Oct 7	2022 Jul 24	Necropsy	High	4.3.3
	*V. variegata* (2)	F	PBZT	2019 Oct 7	2022 Aug 20	Necropsy	High	4.3.4
	*V. variegata* (3)	M	Palmarium Lemur Reserve, Toamasina	2015	2022 Sep 12	Necropsy	High	4.3.3

*PBZT, Botanical and Zoological Park of Tsimbazaza.

Animals that tested positive for *M. tuberculosis* (n = 10) were housed at the PBZT in Antananarivo, Madagascar. Infected animals displayed clinical signs for extended periods, including lethargy, anorexia, vomiting, and fever, leading to death. Necropsies of 4 animals revealed massive lymphohematogenous dissemination in black and white ruffed lemurs (*Varecia variegata*)*,* including nodules, lesions, and white foci noted in lungs, kidneys, spleen, and mesentery (Figure). Caseous, inflamed, and necrotic lymph nodes, and hemorrhages were also present (Appendix Figure 1). Infected, living animals (n = 6) were sampled by using oropharyngeal swabs or bronchoalveolar lavage. No information regarding the presence of *M. tuberculosis* in PBZT staff could be obtained.

**Figure:**
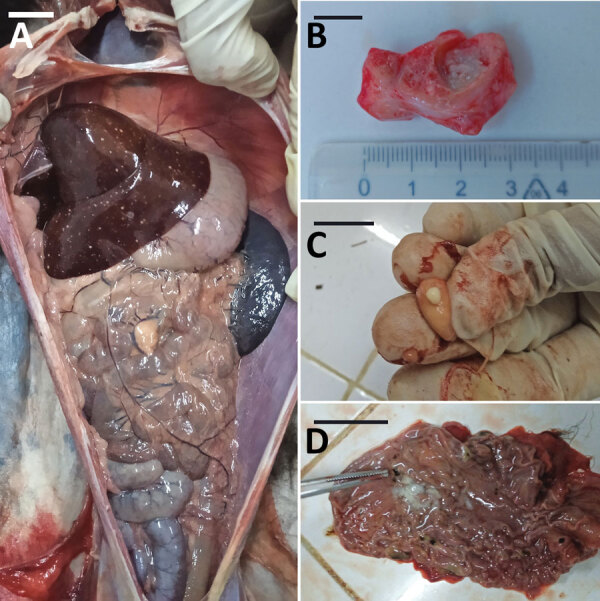
Necropsy images of 2 lemurs (detailed in the Table), both *Varecia variegata*, from a study of *Mycobacterium tuberculosis* complex–positive animals at the Botanical and Zoological Park of Tsimbazaza, Madagascar, in 2022. A) Body cavity with nodules and white spots in liver (animal 3). B) Tracheobronchial caseation of lymph nodes (animal 1). C) Tracheobronchial caseation of lymph nodes (animal 1). D) Black spots in stomach mucosa (animal 3). Scale bars indicate 1 cm.

We confirmed *M. tuberculosis* infection by PCR on the clinical samples using the GeneXpert MTB/RIF Ultra assay (Cepheid, https://www.cepheid.com) and culture on BACTEC-MGIT liquid media (*8*). We conducted whole-genome sequencing on extracted DNA from culture isolates by using MinION (Oxford Nanopore Technologies; https://nanoporetech.com) and NovaSeq PE150 (Illumina, https://www.illumina.com/) sequencing platforms. We mapped decontaminated sequencing reads to the *M. tuberculosis* H37Rv reference genome (accession no. NC_000962.3). Lineage typing based on single-nucleotide polymorphisms revealed all isolates cluster to lineage 4.3.3. (Euro-American lineage, Latin American sublineage) (*9*) and have a maximum distance of 2 single-nucleotide polymorphisms (Appendix Figures 2, 3). GeneXpert MTB/RIF Ultra and sequencing resistance prediction from cultures of clinical isolates did not show any rifampin resistance. 

We deposited all the sequence data used in this study into the National Center for Biotechnology Information Sequence Read Archive (accession no. PRJNA659624). We processed BACTEC-MGIT cultures at the National Reference TB Laboratory at the Institut Pasteur de Madagascar in Antananarivo. For every clinical isolate batch on BACTEC-MGIT culture, we used a positive-control mycobacteria growth indicator tube containing the laboratory reference stain h37Rv and a negative-control tube with the decontamination phosphate buffer. The National Reference TB Laboratory is externally certified twice annually for quality assurance and competency testing on BACTEC-MGIT culture.

Lineage 4 is both the most geographically widespread tuberculosis lineage and the most prevalent in persons residing in Antananarivo (*10*). Primates and other wildlife can contract *Mycobacterium* from humans (*5*). Given that the whole genome sequences from animals in the PBZT have a low maximum distance (2 single-nucleotide polymorphisms) and that the human isolates with the same sublineage L 4.3.3 were found in close proximity to the PBZT around the same time period, there is a possibility that an infected human transmitted the disease to multiple animals. However, it is also possible that interspecies transmission might have occurred, although it is not known whether lemurs or fossas are reservoir hosts for *M. tuberculosis*. If they are, transmission to wild lemurs and other endemic wildlife could pose a threat (*6*) because captive wild animals are sometimes released into forests or live near wild populations (*2*). Moreover, immunocompromised humans may be at risk for *M. tuberculosis* latent or active infection if they are close to diseased animals, such as those at PBZT.

The threat of TB transmission between humans and endangered wildlife, such as lemurs, invokes the need for changes that minimize interactions between humans and wildlife, reducing the chance of new disease outbreaks (*5*,*6*). Recommendations to protect Madagascar wildlife should include no wild capture of animals for captivity, no breeding of wildlife in substandard captive conditions, improved captive care that is comparable to international standards, and humane euthanasia of animals with communicable diseases or disease exposure. We also recommend annual testing (and negative results) for communicable diseases in humans who work in proximity to wildlife and no-contact restrictions for the public and wildlife. Thoese recommendations are consistent with the guidelines of the American Association of Zoo Veterinarians.

Appendix Additional information on tuberculosis in lemurs and a fossa at national zoo, Madagascar, 2022.
