# Supplementary material for: Tuberculosis in Lemurs and a Fossa at National Zoo, Madagascar, 2022
Source: Emerg Infect Dis. 2023 Dec;29(12):2587–9. doi: 10.3201/eid2912.231159 (PMC10683818; doi:10.3201/eid2912.231159)
Supplement: Appendix — Additional information on tuberculosis in lemurs and a fossa at national zoo, Madagascar, 2022. [file 23-1159-Techapp-s1.pdf]

Article DOI: http://doi.org/10.3201/eid2912.231159

EID cannot ensure accessibility for supplementary materials supplied by authors. Readers who have difficulty accessing supplementary content should contact the authors for assistance.

Tuberculosis in Lemurs and a Fossa at National Zoo, Madagascar, 2022

Appendix

Appendix Table. International Union for the Conservation of Nature Red List Status population trend and assessment for species diagnosed with *Mycobacterium tuberculosis* complex at the Botanical and Zoological Park of Tsimbazaza, Madagascar, in 2022.

Species	Assessment	Population trend
Cryptoprocta ferox	Vulnerable	
Eulemur flavifrons	Critically Endangered	
E. fulvus	Vulnerable	
E. rufus	Vulnerable	Decreasing
Lemur catta	Endangered	
Propithecus coquereli	Critically Endangered	
Varecia variegata	Critically Endangered	

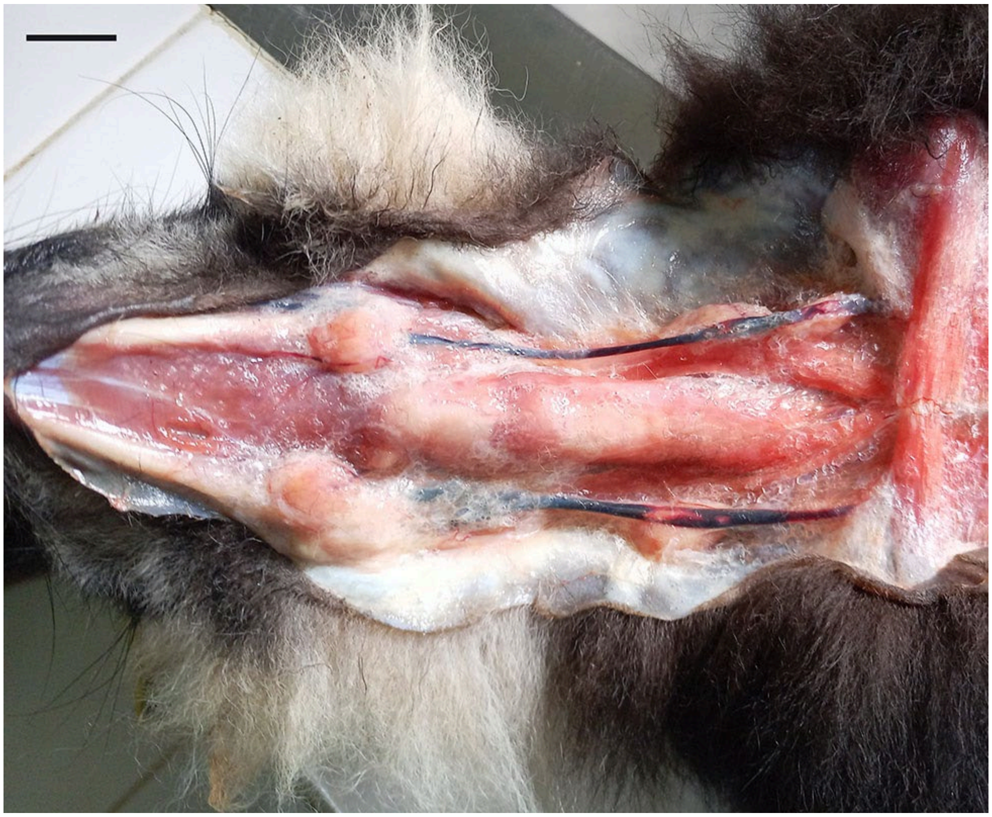

Appendix Figure 1. Inflamed submandibular lymph nodules in the neck/throat region of *Varecia variegata* (individual 1).

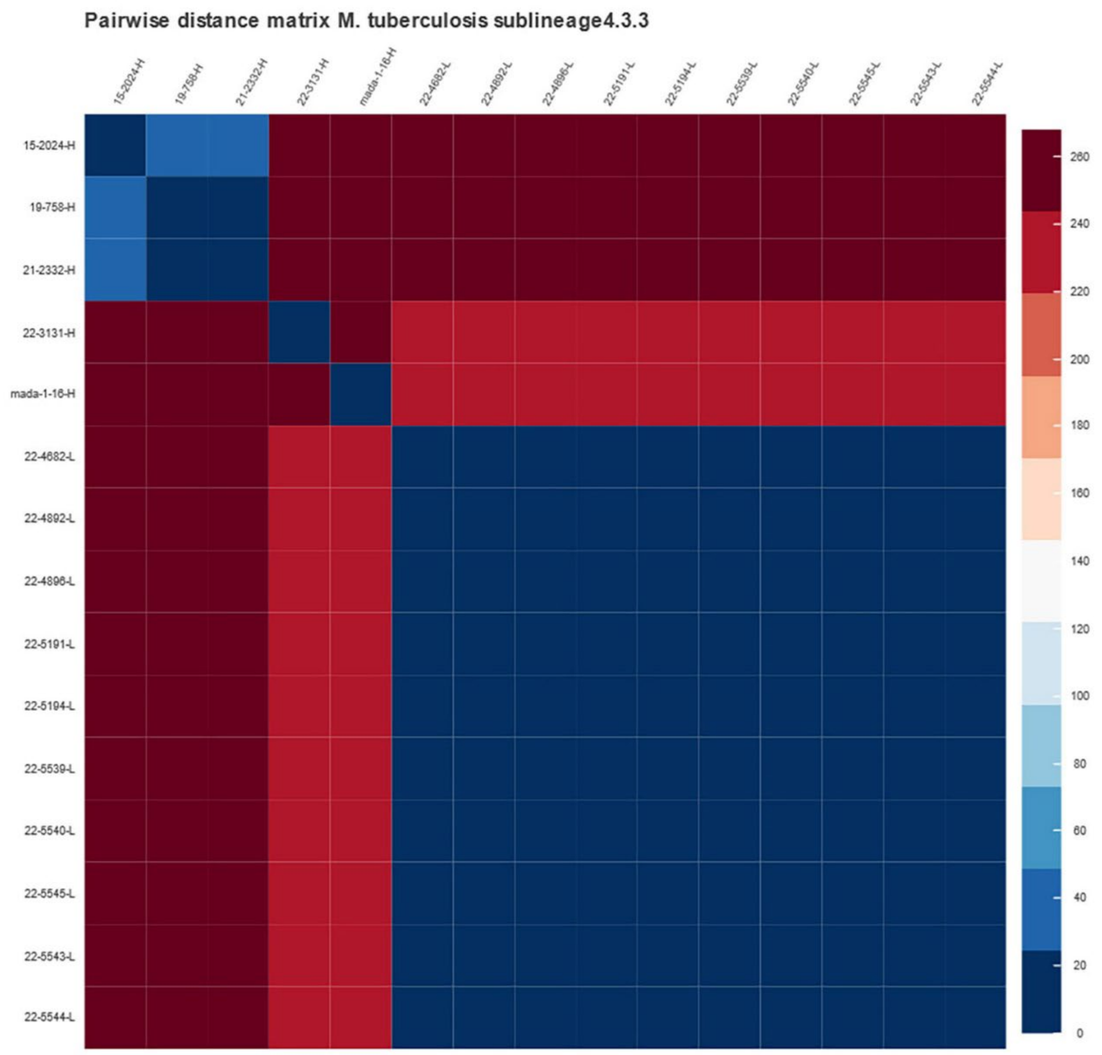

Appendix Figure 2. Pairwise distance matrix of 50 *Mycobacterium tuberculosis* sublineage 4.3.3 isolated from human (H) and lemur (L). Heatmap built with snp-dists 0.8.2.

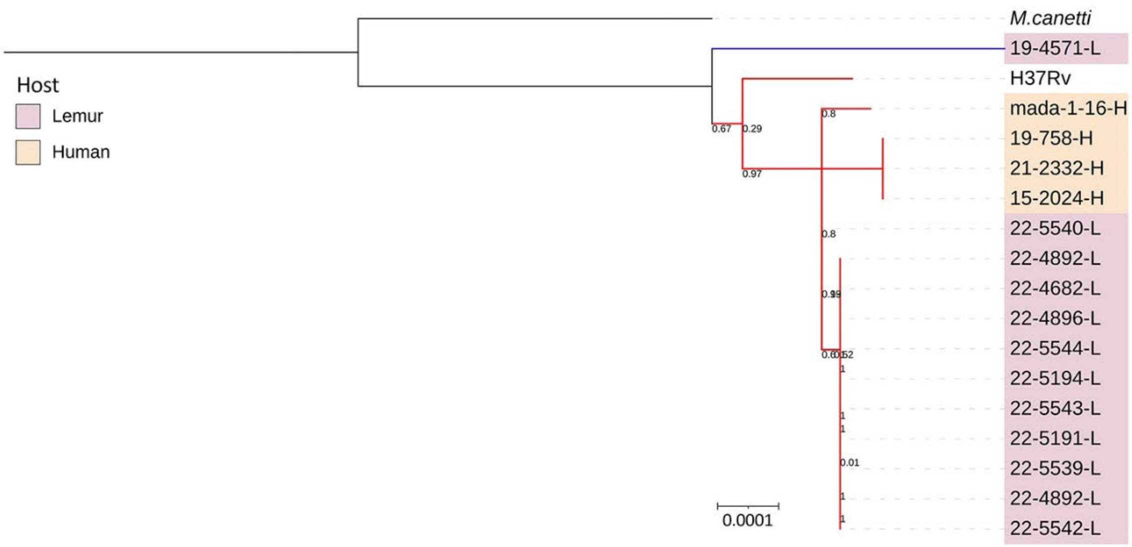

Appendix Figure 3. SNP-based phylogenetic tree of *Mycobacterium tuberculosis* sublineage 4.3.3 (red branches) isolated from lemur in this study, human strains and lineage 3 (blue branch) that was isolated from lemur have been obtained in previous studies. H37Rv (GenBank accession no. NC_000962.3) was used as a reference genome, the tree is rooted in *M. canetti* and designed with Parsnip Software version 1.2 (https://cran.r-project.org/). The software visualization is iTOL (https://itol.embl.de/).
